# Notes and News

**Published:** 1890-09-27

**Authors:** 


### Notes and News.

Collected and Communicated.

The Dental Hospital of London, Leicester Square, has received £50 from Mr. Henry Whiting, being the fifth donation of like amount during the past five years.

The Educational Committee of the London Young Women's Christian Association are again issuing a very attractive prospectus of evening classes. Last autumn and winter nearly 2,000 young women availed themselves of these classes, and numerous prizes and certificates were awarded to those students who proved successful in the recent examinations. At about twenty institutes, in all parts of London, classes are held for teaching dress-cutting, book-keeping, shorthand, type-writing, cookery, ambulance, Civil Service preparation, &c. Prizes and certificates are again offered. There are two good gymnasia, and classes for gymnastic drill at other institutes. A prospectus will be sent free on application to the secretary, 16a, Old Cavendish Street, W.

The London Missionary Hospital at Pekin has issued another report which speaks eloquently of the good work done by the institution. The principal work consists in the treatment of out-patients, of whom there were 19,243 in 1889-90, exclusive of visits to out-patients at their own homes. The entire accommodation for in-patients has for some time been fully used, and while the number of applicants steadily increases, the authorities are unable to receive any more patients. They are all drawn from the poorer classes of Chinese, and it is observed that latterly a great change has taken place in their attitude with regard to amputation which has largely increased the number of operations performed. A curious story of filial devotion is told in which the hero deliberately cut off part of the calf of his leg to make soup for his invalid father. Unfortunately the sacrifice was in vain, as the father died, but it is a good thing for the son that the Missionary Hospital at Pekin is in existence, and he is now undergoing treatment there as an in-patient.

Hospital Saturday reports continue to pour in upon us, and it would be difficult to express too warmly our satisfaction at the rapidity with which the movement is becoming national. Malvern is one of the latest recruits to the cause, and its initial effort has been so successful as to render it extremely probable that the collection will become an annual one. The net result of the day's work was £80 2s., which will be divided between the Rural Hospital and the Dispensary. A sermon was preached by the Vicar the following evening, and a special collection was made in aid of the funds of the latter institution, producing a further sum of nearly £35.

The ninety-first annual report of the Sunderland and Bishopwearmouth Infirmary shows that the number of patients treated during the year was 4,582, of whom 1,873 were in-patients and 2,709 out-patients. Of the total income of £7,956 12s. 3d., workmen's subscriptions produced £4,413 6d., a gratifying proof of the interest taken in the institution by the class deriving most benefit from it. The expenditure amounted to £7,125 1s. 8d., as against £5,269 15s. 10d. last year, the increase being explained by there having been more in-patients, and also by the cost of the alterations in the Backhouse wing, rendered necessary by the transference of the children to the Hartley wing.

A new infectious diseases hospital has been opened by the Rural Sanitary Authority of the Bolton Union. At present the institution consists of a single pavilion divided into four wards, and contains twelve beds and two children's cots, but the scheme contemplates the erection of a second pavilion at some future time, when the hospital will have accommodation for twenty-four beds. Mr. John Wardle, the chairman of the Authority, opened the hospital, for which purpose a gold key was presented to him, and after the ceremony the general public were admitted to inspect the buildings. The grounds cover two and a-quarter acres of land, and the structures and sanitary arrangements are said to be upon the most complete and improved principles.

"In loving memory of a dear father and in the hope of fulfilling one of his latest wishes," the Misses Ormerod have built a convalescent home for children at St. Anne's-on-the-Sea. The institution, which has accommodation for forty patients, was open to receive inmates last week, having been formally opened the previous Saturday by Lady Eleanor Cecily Clifton. The home is intended for the accommodation of the children of the poor of Lancashire, and by desire of the founders preference will also be given to children belonging to Todmorden in Yorkshire, where the late Mr. Ormerod lived for many years. The home will be under the charge of the Kilburn sisters, and is entirely dependent for its maintenance upon voluntary contributions.

Stroud did bravely on the occasion of its hospital centenary celebration on the 13th, and of the total amount required to complete the fund for adding a new wing to the institution in commemoration of the anniversary some £300 was realized in the course of the day. In addition to the ladies' collection in the streets there was a monster procession representing the various industries of the town, and swelled by fire-brigades, benefit societies, volunteers, and bands. Stratford Park was thrown open in the afternoon, and a flower show and promenade concert was held in the grounds, and in the evening there was a concert in the Subscription Rooms, while outside the streets were illuminated; and there was a second procession of cyclists, whose machines were decorated with lanterns, and of the fire-brigade, whose escape took passengers "up the ladder and down the shoot" for twopence, the proceeds being, of course, for the hospital. The foundation-stone of the new wing was laid by Mr. Holloway, the member for the division.

